# Seed Quality Traits Can Be Predicted with High Accuracy in *Brassica napus* Using Genomic Data

**DOI:** 10.1371/journal.pone.0166624

**Published:** 2016-11-23

**Authors:** Jun Zou, Yusheng Zhao, Peifa Liu, Lei Shi, Xiaohua Wang, Meng Wang, Jinling Meng, Jochen Christoph Reif

**Affiliations:** 1 National Key Laboratory of Crop Genetic Improvement, Huazhong Agricultural University, Wuhan, China; 2 Leibniz Institute of Plant Genetics and Crop Plant Research (IPK), Gatersleben, Germany; New South Wales Department of Primary Industries, AUSTRALIA

## Abstract

Improving seed oil yield and quality are central targets in rapeseed (*Brassica napus*) breeding. The primary goal of our study was to examine and compare the potential and the limits of marker-assisted selection and genome-wide prediction of six important seed quality traits of *B*. *napus*. Our study is based on a bi-parental population comprising 202 doubled haploid lines and a diverse validation set including 117 *B*. *napus* inbred lines derived from interspecific crosses between *B*. *rapa* and *B*. *carinata*. We used phenotypic data for seed oil, protein, erucic acid, linolenic acid, stearic acid, and glucosinolate content. All lines were genotyped with a 60k SNP array. We performed five-fold cross-validations in combination with linkage mapping and four genome-wide prediction approaches in the bi-parental population. Quantitative trait loci (QTL) with large effects were detected for erucic acid, stearic acid, and glucosinolate content, blazing the trail for marker-assisted selection. Despite substantial differences in the complexity of the genetic architecture of the six traits, genome-wide prediction models had only minor impacts on the prediction accuracies. We evaluated the effects of training population size, marker density and phenotyping intensity on the prediction accuracy. The prediction accuracy in the independent and genetically very distinct validation set still amounted to 0.14 for protein content and 0.17 for oil content reflecting the utility of the developed calibration models even in very diverse backgrounds.

## Introduction

Rapeseed *(Brassica napus* L.) is one of the most important oilseed crops worldwide [1]. The breeding goal for rapeseed is high oil yield coupled with excellent oil quality [2–4]. The latter is mainly driven by the composition of the fatty acid components of erucic acid (C22:1), stearic acid (C18:0), oleic acid (C18:1), linoleic acid (C18:2), and linolenic acid (C18:3) [2, 3, 5]. Moreover, protein and glucosinolate content determine to a large extent the quality of the rapeseed meal [6–8]. All of these seed traits are influenced by the environment [9–11], and their precise estimation requires phenotyping in replicated multi-environmental field trials. Moreover, measuring quality traits in rapeseed is often labor-intensive. Therefore, quality traits are interesting targets for genomic-assisted crop improvement.

Genomic-assisted crop improvement can either be based on marker-assisted selection [12] or genome-wide predictions [12, 13]. In marker-assisted selection, the performance of individuals is predicted using a few diagnostic markers associated with the traits under consideration [14]. In contrast, genome-wide prediction exploits many markers without performing marker-specific significance tests [15]. The accuracy of marker-assisted selection and genome-wide predictions depends on the genetic architecture underlying the traits under consideration. Marker-assisted selection is most effective if the trait is controlled by a few genes with large effects. If the genetic architecture is complex, quantitative trait loci (QTL) detection is not reliable and genome-wide prediction is more powerful [16].

The presence of QTL underlying quality traits in rapeseed has been investigated in linkage and linkage disequilibrium mapping studies [1, 3, 9–11, 17–28]. Accumulated information of the QTL accounting for seed quality traits such as seed fatty acid has also been identified in other *Brassica* species, such as *B*. *oleracea* and *B*. *juncea* [29, 30], which could provide reference for the comparison between species. However, linkage and linkage disequilibrium mapping, are often afflicted by upwards biased estimates in terms of the proportion of genotypic variance explained by QTL. Therefore, cross- or independent validations have been suggested to obtain unbiased estimates of QTL effects but have been applied only in a limited number of studies in rapeseed [31, 32].

The potential and limits of genome-wide predictions have been examined for several major crops, such as barley [33], wheat [15, 34–36], maize [37–42], rice [43], sunflower [44], forage plants [45], sugar beet [46, 47], and soybean [48, 49]. The results underlined the potential of genome-wide prediction as a powerful tool to accelerate selection gain in plant breeding. Recent studies in rapeseed also highlighted the potential of genome-wide prediction of flowering time [31, 50, 51], plant height, protein content, oil content, glucosinolate content, grain yield [31, 51]. Nevertheless, the benefits of genome-wide prediction compared to marker-assisted selection have not been examined in rapeseed. Moreover, the potential to exploit epistasis to predict seed quality traits has not been investigated, although previous studies suggested that epistatic interactions were important for fatty acid metabolism [11].

This study is based on a published dataset from the bi-parental TN DH population comprising 202 DH lines, which has been intensively used to study the genetic architecture of important agronomic traits [9–11, 22, 23] and were genotyped with an Infinium 60K-SNP array [52] being extensively used in *Brassica* [24, 53, 54]. The two parents of the TN DH mapping population originated from the European and Chinese genepools and have been used widely for rapeseed breeding programs in both target regions. Our objectives were to (i) test for the presence of QTL exhibiting reliable and large effects using five-fold cross-validations, (ii) investigate the effect of the genetic architecture on the superiority of different genome-wide prediction models, (iii) examine the potential to improve the prediction accuracy by modeling digenic epistatic effects, (iv) validate the prediction accuracy in a genetically independent population, and (v) discuss the consequences for implementing genome-wide predictions in applied rapeseed breeding programs.

## Materials and Methods

### Plant materials and field trials

A bi-parental DH population of *B*. *napus* denoted as TN DH has been developed, comprising 202 unique lines [22]. The DH lines were derived from a microspore culture based on the F_1_ cross between Tapidor and Ningyou7. The parent Tapidor is a European winter cultivar with low erucic acid and glucosinolate content in the seeds. The parent Ningyou7 is a Chinese semi-winter cultivar with high erucic acid and glucosinolate content in seeds. The TN DH mapping population along with its two parents was grown in 11 winter and semi-winter ecotype environments (S1 Table). The phenotypic data was generated and used in a previous linkage mapping study, which was based on a limited set of markers [9–11, 22, 23]. The experimental design was a randomized complete block design with 3 replications. Every plot comprised three rows with a total plot size of 3.0 to 4.0 m^2^.

Phenotypic data was collected for six important seed quality traits for each DH line and parent: seed oil content (%) and protein content (%), which were separately defined as the percentages of the oil and protein in the total seed dry weight, respectively; three important components of the fatty acid in the seed oil: the erucic acid content (%), the linolenic acid content (%), and the stearic acid content (%); and the content of glucosinolates in the total seed dry weight (µmol/g). The quality traits were determined based on near infrared reflectance spectroscopy measuring three technical and three biological replicates. The details of the phenotyping are outlined in detail in previous studies [10, 11, 22].

A total of 117 genetically independent *B*. *napus* inbred lines were used in this study for validating the prediction accuracy based on the TN DH population. The validation population was developed based on hundreds of crosses between *B*. *rapa* and *B*. *carinata* accessions [55, 56]. The validation population was grown in one semi-winter environment (Wuhan, China) in 2013–2014 in a trial with three replicates. Every plot comprised two rows with a total plot size of 2.0 to 3.0 m^2^. Seed oil content and protein content was measured using the same method as that used for the TN DH population.

### Phenotypic data analyses

The best linear unbiased estimates (BLUEs) of phenotypic values and variance components were estimated by the following linear mixed model using ASREML-R software [57]:

Traits∼Genotype+Environment+Genotype:Environment+Environment:Rep.

The genotype effects were treated as fixed effects and the other effects were treated as random. To estimate variance components, all effects were treated as random. Broad-sense heritability was calculated as the ratio of genotypic to phenotypic variance:

$$H^{2}=\frac{\sigma_{G}^{2}}{\sigma_{G}^{2}+\frac{\sigma_{GxE}^{2}}{N_{E}}+\frac{\sigma_{E}^{2}}{N_{E}*N_{R}}},$$

where N_E_ refers to the number of environments, N_R_ is the average number of replications per location, $\sigma_{G}^{2}$ is the genotypic variance, $\sigma_{GxE}^{2}$ is the variance of genotype times environment interaction, and $\sigma_{E}^{2}$ refers to the error variance.

### Genotypic data analyses

The 202 DH lines of the TN DH population and the two parents were previously fingerprinted using a 60k SNP array based on an Illumina Infinium assay [52]. Quality control was performed and those markers have been removed which are either monomorphic, have missing values of >5%, a minor allele frequency <5%, or degree of heterozygosity >5% in the DH population. After applying the quality check outlined above, 180 DH lines with 13,678 high-quality SNP markers remained. By aligning the marker sequence of the 13,678 SNPs to the reference “Darmor-bzh” genome of *B*. *napus* version 4.1[58] via BLAST analysis, 9,628 SNP markers could be assigned a unique physical position in the genome with the parameters of 100% alignment, E value <10^−20^ and mismatch <2 (S2 Table). After removing redundant SNPs in full linkage disequilibrium (LD), 1,527 markers representing recombination loci (referred to as representative markers) remained (S2 Table). The 1,527 representative markers included 1,052 representative markers from 1,052 genetic bins and 475 single markers. From each of the genetic bins, one marker with the least missing rate and the best available physical alignment position was selected as representative marker. In this way, a total of 1,527 representative markers were obtained and used for the subsequent analysis. Pairwise LD between markers was calculated as the squared Pearson moment correlation coefficient using R package genetics [59]. The 117 lines of the validation population were genotyped using the same SNP array and the 1,527 representative markers selected in the TN DH population were used for prediction.

### QTL mapping and genome-wide prediction

For the QTL mapping, the SNP markers were coded according to the F_∞_ metric [60]. The genome-wide QTL mapping method is based on the inclusion of cofactors [7] obtained by stepwise multiple linear regressions using the Bayesian information criterion [61]. The genome-wide scan was conducted comparing the full model comprising the SNP and all cofactors versus a reduced model including only cofactors. We used a false-discovery rate (FDR) of P<0.1 to test for significance. The proportion of the phenotypic variance explained (PVE) by all QTLs, was estimated using the adjusted R^2^ values fitting a multiple regression [62].

We performed a five-fold cross-validation of the QTL mapping in which the total population of 180 DH lines was randomly divided into two groups with 100 replications according to the ratio of 4:1 (one group with 144 lines and the other group with 36 lines). One hundred and forty-four lines were used as the training set and the remaining 36 were used as the test set. QTL mapping was performed in each training set and estimated QTL effects were used to predict the genetic values of the lines of the test set. The prediction accuracy was defined as the correlation between the predicted and observed phenotypic values standardized with the square root of the heritability.

For the genome-wide prediction, four different models were used in this study. We implemented three methods exploiting the additive marker effect: genomic best linear unbiased prediction (GBLUP), ridge regression best linear unbiased prediction (RR-BLUP) [63], and BayesCπ [64]. To accelerate computation speed and eliminate the impact of LD on the prediction accuracy of BayesCπ, we removed SNPs with *r*^2^>0.95. For BayesCπ, the Gibbs sampling ran 20,000 times, and the first 6,000 cycles were used as burn in. We also implemented an extended GBLUP model denoted as EG-BLUP, which models digenic epistatic effects as well as additive effects [65]. The accuracies of all these genome-wide prediction methods were determined based on the adjusted entry means for the 180 genotypes applying five-fold cross-validation. Details of the implementation of the models have been described elsewhere [41, 42, 65]. We performed 100 cross-validation runs and estimated the accuracy as the Pearson correlation coefficient between predicted and observed values standardized with the square root of the heritability.

To evaluate the dependence of prediction accuracy on training set size, we applied cross-validation with randomly selected subsets of n (n = 48, 80, 112, 144) lines from the full data to form the training set and used the remaining lines as the test set. To evaluate the dependence of prediction accuracy on marker density, we selected subsets of m (m = 100, 1,000, 5,000, 13,678) evenly distributed markers from the full dataset and applied five-fold cross-validations using all 180 lines. The sampling procedure was randomly repeated 100 times for each scheme, and the prediction accuracies were averaged across the 100 cross-validation runs. We focused in the above outlined analyses of sampling of marker subsets and training set sizes on the traits seed oil content and protein content. The traits were selected because oil content was evaluated in a large number of 11 environments and protein content exhibited a high heritability.

We also evaluated the prediction accuracy using an independent validation population. The marker effects were estimated based on RR-BLUP and the TN DH population. Marker effects were used to predict the performance of the 117 individuals of the validation population. The prediction accuracy was again estimated as the Pearson correlation coefficient between predicted and observed values standardized with the square root of the heritability. Heritability was estimated using the variance components estimated for the TN DH population.

## Results

### Intensive field evaluation of the TN DH population resulted in high-quality phenotypic data

**Fig 1:**
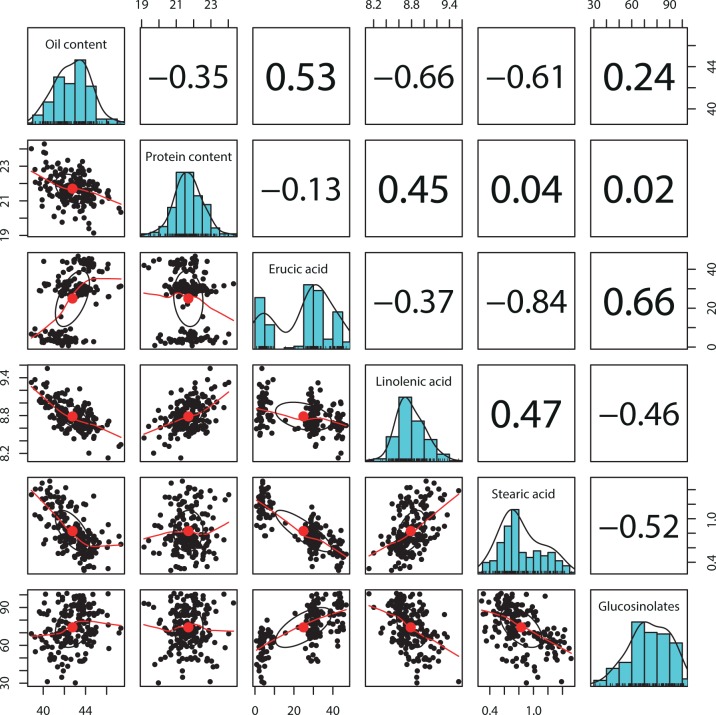
Distributions and pairwise correlations for Best Linear Unbiased Estimates of six seed traits evaluated for 202 lines of the TN DH population in multi-environmental field trials. All correlations passed significance tests with P-values less than 0.001 except for the correlation between protein content and erucic acid, glucosinolates, and stearic acid content.

We combined the information on seed protein content with previously published data for other seed quality traits of the TN DH population. We observed a wide variation of BLUEs approximating a normal distribution for most traits, except for erucic acid content (Fig 1, S3 Table). The analyses across environments revealed significant (P<0.001) variances for genotypes, environments, and interactions between genotypes and environments (Table 1). Broad-sense heritability estimates were high for the six traits, ranging from 0.81 for protein content to 0.98 for erucic acid content. Consequently, the intensive phenotyping resulted in high-quality data representing an excellent source for dissecting the genetic basis of the six traits.

**Table 1:** Estimates of variance components (σ^2^) and broad-sense heritability (h^2^) for the TN DH population with 202 lines evaluated for six seed traits in multi-environmental field trials.

Source*/Traits	Oil content	Protein content	Erucic acid content	Linolenic acid content	Stearic acid content	Glucosinolate content
$\sigma_{G}^{2}$	2.64	0.53	198.19	0.04	0.08	229.38
$\sigma_{G\times E}^{2}$	0.71	0.42	11.84	0.01	0	120.82
$\sigma_{E}^{2}$	1.17	0.61	7.78	0.02	0.01	54.55
Heritability	0.96	0.81	0.98	0.82	0.94	0.9
Mean	42.76	21.69	24.91	8.79	0.81	74.16
Range	38.87–47.35	19.13–24.3	0.77–46.76	8.13–9.55	0.27–1.50	30.31–101.17
Nr. of environments	11	5	5	2	2	6

*All variances pass a significance test with P values less than 0.001.

In total, 80% of the pairwise trait comparisons were significantly (P<0.001) associated with Pearson moment correlation coefficients ranging from -0.84 between erucic acid content and stearic acid content to 0.66 between erucic acid content and glucosinolate content (Fig 1). Interestingly, protein content was only poorly associated with erucic acid, glucosinolate, and stearic acid content. This lack of associations points to independent biochemical pathways and genes controlling the two classes of traits.

### Large differences in the complexity of the genetic architecture of the six seed quality traits

**Fig 2:**
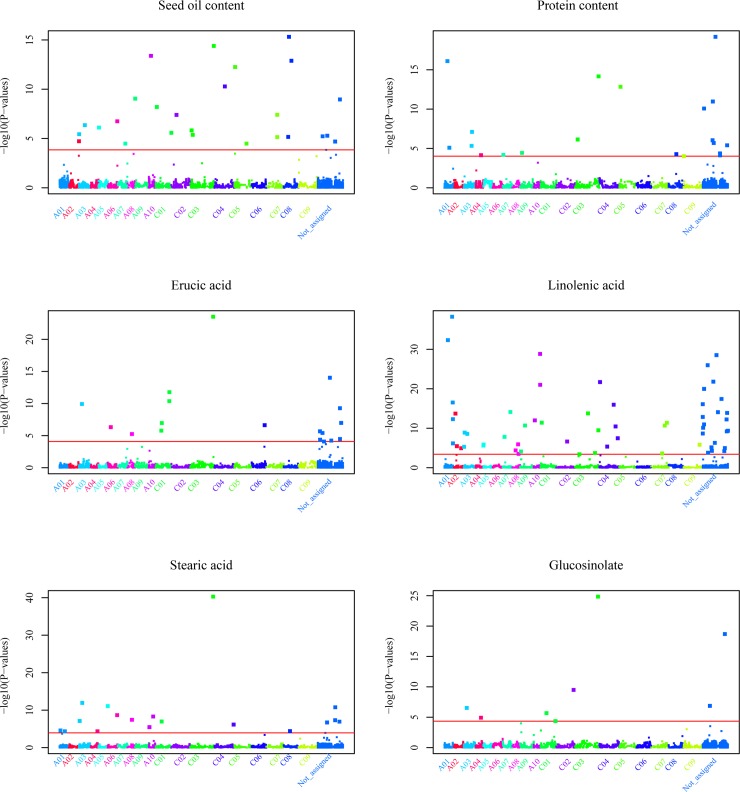
Manhattan plots based on composite interval QTL mapping for the six seed quality traits. The x-axis represents the corresponding physical position of each SNP of the 13,678 SNPs across the genome from chromosome A01 to A10 and C01 to C09. Those markers without unique alignment to the reference genome were arranged in the axis noted as “not assigned”. The Y-axis represents the corresponding false-discovery rate (FDR) of each QTL indicating the significance for QTL calling. The PVE, i.e. proportion of the phenotypic variance explained by each QTL, is listed in Table 2.

Altogether, 151 SNP markers passed the FDR significance level of P<0.1 in the genome-wide QTL mapping scan (Figs 2 and S1). The QTL numbers for the six traits ranged from 8 to 59 and were distributed across 19 chromosomes of *B*. *napus*. Phenotypic variance explained by a single putative QTL exceeded 5% for 27 SNPs and reached 45% for a QTL located on chromosome C03 controlling erucic acid content (Table 2). A second major QTL was detected on chromosome A08 for erucic acid content, explaining 31% of the phenotypic variance. However, the majority of the QTLs, especially those influencing oil and protein content, exhibited only minor effects. Among the detected QTLs, seven were putative pleiotropic QTLs influencing two traits. For instance, the marker “Bn-scaff_15794_1-p347392”, which was physically aligned to C03 and detected as a putative pleiotropic QTL, explained 26% and 45% of the phenotypic variance for stearic acid and erucic acid concentration, respectively.

**Table 2:** Significant marker-trait associations and the proportion of explained phenotypic variance (PVE) detected in a genome-wide association mapping approach for six quality traits of TN DH population.

Trait	No.	Marker	P values	PVE	Genetic	Chr.	Physical	Detected in previous studies
					bin code^1^		position (bp) ^2^	
Oil content	1	Bn-A10-p5869175	4.09E-14	4.27	612	A10	5499050	TN-qOC-A10-1 (Jiang et al. 2014)^[^^10^^]^
	2	Bn-A09-p739088	9.01E-10	2.96	551	A09	131541	
	3	Bn-A07-p16379135	3.29E-05	0.01	485	A07	18348848	SG-qOC-A7 (Zhao et al. 2012)^[^^66^^]^
	4	Bn-A07-p15802174	5.96E-06	0.7	single marker	A07	NA^3^	
	5	Bn-A04-p1684695	5.22E-11	5.86	806	C04	25032235	
	6	Bn-A01-p27774666	2.64E-06	0.5	single marker	C01	38105589	
	7	Bn-scaff_15695_1-p294894	3.31E-05	0.25	854	C05	29927748	
	8	Bn-scaff_16361_1-p930064	0.000142	1.23	956	C08	NA^3^	
	9	Bn-scaff_20942_1-p440106	5.21E-06	9.49	694	C02	NA^3^	
	10	Bn-scaff_17637_1-p204439	6.89E-06	2.74	945	C08	12326547	
	11	Bn-scaff_16565_1-p1169320	4.06E-08	1.18	698	C02	12445051	TN-qOC-C2-2 (Jiang et al. 2014)^[^^10^^]^
	12	Bn-scaff_15838_1-p2253503	6.36E-09	2.94	660	C01	2629345	TN-qOC-C1-1 (Jiang et al. 2014)^[^^10^^]^
	13	Bn-A05-p1308471	7.76E-07	0.06	309	A05	1423576	SG-qOC-A5 (Zhao et al. 2012)^[^^66^^]^
	14	Bn-Scaffold000217-p20168	2.05E-05	0.37	361	C05	NA^3^	
	15	Bn-scaff_20901_1-p1705574	5.59E-13	4.91	839	C05	2309449	
	16	Bn-scaff_23761_1-p249628	4.09E-15	16.44	single marker	C03	57481703	TN-qOC-C3-3 (Jiang et al. 2014)^[^^10^^]^
	17	Bn-A02-p27799727	1.92E-05	0.93	139	A02	24756539	DY-qOC-A2-2 (Delourme et al. 2006)^[^^1^^]^; Z5-qOC-A2-1 (Sun et al. 2012)^[^^67^^]^
	18	Bn-scaff_16231_1-p2213239	1.30E-13	5.75	949	C08	20090489	
	19	Bn-A03-p15397187	4.40E-07	3.81	195	A03	14446606	TN-qOC-A3-3 (Jiang et al. 2014)^[^^10^^]^
	20	Bn-scaff_16545_1-p238397	4.80E-16	6.43	508	C08	14155605	
	21	Bn-A06-p7949147	1.09E-09	1.35	388	A06	NA^3^	
	22	Bn-scaff_16130_1-p1013445	3.94E-08	1.79	911	C07	28755038	
	23	Bn-scaff_16130_1-p1039452	7.23E-06	2.28	911	C07	28772215	
	24	Bn-A06-p24132842	1.79E-07	2.49	421	A06	23129285	Z5-qOC-A6-1 (Sun et al. 2012)^[^^67^^]^
	25	Bn-scaff_22728_1-p357789	4.28E-06	0.88	160	C03	6154024	TN-qOC-C3-3 (Jiang et al. 2014)^[^^10^^]^; OIL.C3.s.1(Niklas Körber et al.2016)^[^^68^^]^
	26	Bn-A03-p764274	3.67E-06	0.06	141	A03	632475	
	27	Bn-scaff_18936_1-p890286	1.50E-06	1.48	731	C03	3419666	OIL.C3.s.1(Niklas Körber et al. 2016)^[^^68^^]^
Protein content	1	Bn-scaff_15838_3-p256767	8.37E-11	7.13	121	A02	NA^3^	
	2	Bn-A03-p21225846	7.85E-08	0.08	211	A03	19974471	
	3	Bn-A04-p12670129	7.18E-05	1.96	269	A04	13394800	qThrC-4-2(Xu et al.2015)^[^^69^^]^
	4	Bn-A03-p20150479	4.68E-06	2.65	209	A03	19014117	
	5	Bn-scaff_16361_1-p300435	8.14E-06	0.5	63	A01	11871025	
	6	Bn-A09-p5190180	3.69E-05	0.02	556	A09	4862135	
	7	Bn-scaff_17526_1-p860459	9.60E-05	5.21	977	C09	1679866	qMetC-19-9(Xu et al.2015)^[^^69^^]^
	8	Bn-scaff_16449_1-p251526	9.17E-07	0.42	709	C02	NA^3^	
	9	Bn-A09-p33595011	1.08E-11	2.85	single marker	NA^3^	NA^3^	
	10	Bn-A01-p8058255	7.87E-17	9.49	50	A01	7238500	qPC-1(Huang et al.2016)^[^^70^^]^
	11	Bn-A09-p15975138	2.03E-06	1.91	567	A09	NA^3^	
	12	Bn-scaff_17119_1-p349622	6.35E-20	10.98	778	C03	NA^3^	
	13	Bn-scaff_17119_1-p414142	6.82E-15	5.37	778	C03	57158030	
	14	Bn-scaff_27815_1-p367403	6.45E-05	2.65	431	A07	1626003	
	15	Bn-A01-p27125649	4.28E-05	1.35	87	A01	NA^3^	
	16	Bn-Scaffold000217-p38276	7.56E-05	1.78	361	C05	NA^3^	
	17	Bn-scaff_20901_1-p647270	1.44E-13	10.05	840	C05	3389245	qAlaC-15-4 (Wen et al.2015)^[^^71^^]^
	18	Bn-scaff_16231_1-p2213239	5.32E-05	8.59	949	C08	20090489	
	19	Bn-scaff_23799_1-p6782	3.97E-06	1.76	single marker	NA^3^	NA^3^	
	20	Bn-scaff_22728_1-p349077	7.20E-07	2.77	160	C03	6162734	qMetC-13-6 (Xu et al.2015)^[^^69^^]^
Erucic acid	1	Bn-scaff_15803_1-p800874	1.11E-07	0.36	58	C01	14815203	
	2	Bn-scaff_15747_1-p167954	2.16E-06	0.08	675	C01	NA^3^	
	3	Bn-scaff_19614_1-p36023	1.66E-06	0.11	675	C01	13532546	
	4	Bn-A03-p24897111	4.39E-05	1.08	223	A03	NA^3^	
	5	Bn-scaff_18039_1-p206042	4.22E-11	2.08	682	C01	33006005	
	6	Bn-scaff_15844_1-p119216	1.68E-12	0.63	single marker	C01	33345268	
	7	Bn-A03-p23609934	3.90E-06	0.1	219	A03	NA^3^	
	8	Bn-A01-p3664698	8.21E-05	1.95	33	A01	NA^3^	
	9	Bn-scaff_16397_1-p21961	2.35E-07	0.38	885	C06	32884939	ERA.C6.s.1(Niklas Körber et al.2016)^[^^68^^]^
	10	Bn-scaff_15794_1-p347392	2.97E-24	45.35	775	C03	55942754	qC3-3(Wang et al.2015)^[^^11^^]^
	11	Bn-A09-p19718581	9.53E-15	0.63	568	A09	NA^3^	
	12	Bn-scaff_17984_1-p123918	5.88E-05	3.54	569	A09	NA^3^	
	13	Bn-A08-p13221380	5.64E-06	31.31	510	A08	10967853	qA8-5(Wang et al.2015)^[^^11^^]^
	14	Bn-C14160250-p3687	5.39E-10	0.98	509	A08	NA^3^	
	15	Bn-A06-p7636729	4.83E-07	0.11	388	A06	7278355	
	16	Bn-A06-p7459428	3.40E-05	0.69	single marker	A06	NA^3^	
	17	Bn-A03-p14811204	1.05E-07	0.03	191	A03	NA^3^	
	18	Bn-A03-p8177695	1.19E-10	1.91	173	A03	7472584	
Linoleic acid	1	Bn-A02-p1890913	1.40E-13	0.23	655	NA^3^	NA^3^	
	2	Bn-A02-p2451470	7.90E-17	1.76	655	NA^3^	NA^3^	
	3	Bn-A02-p7105435	1.89E-14	0.22	single marker	A02	4150237	
	4	Bn-A10-p510846	1.01E-12	8.55	610	A10	2998077	
	5	Bn-A10-p1193336	2.13E-09	3.29	609	A10	NA^3^	
	6	Bn-A10-p2092612	7.56E-11	0.11	single marker	A10	NA^3^	
	7	Bn-A09-p3546619	1.99E-22	4.68	single marker	C04	1322839	
	8	Bn-A02-p10850012	3.55E-06	9.07	118	A02	7665679	
	9	Bn-A02-p12145607	1.07E-11	0.12	123	A02	NA^3^	
	10	Bn-A09-p20863459	1.06E-20	1.99	single marker	NA^3^	NA^3^	
	11	Bn-A09-p1631944	7.56E-05	5.13	554	A09	2294691	LIA.A9.w.1(Niklas Körber et al.2016)^[^^68^^]^
	12	Bn-scaff_22749_1-p250319	2.31E-07	0.6	129	C02	26414418	
	13	Bn-A07-p16846624	7.65E-15	1.68	485	A07	18775516	
	14	Bn-A01-p27968584	1.06E-26	0.93	single marker	NA^3^	NA^3^	
	15	Bn-A02-p18438691	0.000162	3.28	single marker	A02	NA^3^	
	16	Bn-A02-p19070958	1.17E-05	1.08	131	A02	18106292	
	17	Bn-scaff_18855_1-p795432	1.72E-14	0.06	757	C03	31378910	
	18	Bn-scaff_16135_1-p196922	0.00032	0.03	532	A08	15018355	
	19	Bn-A10-p15742689	1.45E-29	2.59	648	A10	15807427	
	20	Bn-A05-p18147040	2.03E-11	1.09	248	A09	12040388	
	21	Bn-scaff_16372_1-p19665	0.000194	1.27	769	C03	48510330	qC3-2(Wang et al.2015)^[^^11^^]^
	22	Bn-scaff_20294_1-p438293	6.85E-06	3.77	887	C06	NA^3^	
	23	Bn-A10-p15442975	9.97E-22	2.37	652	A10	16049734	
	24	Bn-scaff_17799_1-p2773426	1.55E-06	~0.00	990	C09	39884740	
	25	Bn-A09-p33542334	5.40E-05	0.83	single marker	A09	NA^3^	
	26	Bn-A01-p22016353	5.40E-39	5.44	76	A01	18645502	
	27	Bn-A05-p114598	2.39E-06	0.01	single marker	A05	128946	
	28	Bn-A01-p9810552	4.72E-33	0.04	single marker	A01	8432723	
	29	Bn-A01-p8108178	1.57E-22	1.96	50	A01	NA^3^	
	30	Bn-scaff_17821_1-p21053	3.33E-10	3.01	777	C03	56695853	qC3-3(Wang et al.2015)^[^^11^^]^; qC18:2-13-5(Wen et al.2015)^[^^71^]
	31	Bn-A09-p19688476	5.00E-07	0.02	568	A09	NA^3^	
	32	Bn-A05-p472271	1.39E-06	0.37	307	A05	583644	
	33	Bn-scaff_15838_1-p2253503	3.85E-12	7.51	660	C01	2629345	qC18:2-11-3(Wen et al.2015)^[^^71^^]^
	34	Bn-scaff_15585_1-p1020764	3.37E-08	0.96	279	C04	44431942	
	35	Bn-scaff_15676_1-p341508	2.92E-29	2.23	858	C05	NA^3^	
	36	Bn-scaff_19170_1-p1107619	4.39E-06	0.08	10	C04	18803084	
	37	Bn-scaff_19170_1-p588356	8.41E-15	0.85	10	C04	NA^3^	
	38	Bn-A10-p3329131	1.54E-08	3.3	433	A07	4431854	
	39	Bn-Scaffold000164-p120459	6.78E-07	0.22	83	A01	20722278	
	40	Bn-scaff_21956_1-p160710	3.46E-11	0.72	821	C04	39249761	
	41	Bn-scaff_16876_1-p171510	3.75E-18	0.11	817	NA^3^	NA^3^	
	42	Bn-scaff_16876_1-p303006	1.11E-16	0.46	816	C04	34584781	
	43	Bn-A01-p24830111	5.08E-13	0.43	82	A01	20561797	
	44	Bn-A08-p16562035	1.20E-06	0.28	522	A08	14030898	qA8-5(Wang et al.2015)^[^^11^^]^; LIA.A8.w.1(Niklas Körber et al.2016)^[^^68^^]^
	45	Bn-scaff_16069_1-p3780494	4.12E-12	0.04	929	C07	40184750	
	46	Bn-scaff_16545_1-p110342	4.09E-05	0.01	508	A08	7961925	qA8-6(Wang et al.2015)^[^^11^^]^
	47	Bn-scaff_27204_1-p1544	6.30E-05	0.42	1045	C07	NA^3^	
	48	Bn-A08-p11212494	1.06E-05	0.47	509	A08	NA^3^	
	49	Bn-scaff_16130_1-p1039452	0.000253	0.07	911	C07	28772215	
	50	Bn-scaff_15705_1-p1818177	2.15E-11	0.29	918	C07	35089587	
	51	Bn-A02-p2962298	5.75E-13	0.09	single marker	A02	NA^3^	
	52	Bn-A03-p10883930	6.17E-10	0.08	1051	A03	NA^3^	
	53	Bn-scaff_19111_1-p177344	0.000399	0.2	744	C03	10482820	
	54	Bn-A03-p9098773	2.74E-09	0.39	173	A03	8405389	
	55	Bn-scaff_23799_1-p6782	1.32E-14	0.88	single marker	NA^3^	NA^3^	
	56	Bn-A03-p2491346	1.29E-09	0.24	150	A03	2036063	
	57	Bn-A01-p24020451	2.95E-17	0.15	single marker	A01	20087415	
	58	Bn-A03-p764274	5.12E-06	0.73	141	A03	632475	
	59	Bn-A02-p2287712	4.39E-10	1.09	single marker	NA^3^	NA^3^	
Stearic acid	1	Bn-A10-p9436205	5.05E-09	1.5	single marker	A10	10869232	qA10-2(Wang et al.2015)^[^^11^^]^
	2	Bn-A10-p1919293	3.42E-06	0.04	607	A10	1780462	
	3	Bn-scaff_15747_1-p396080	1.09E-07	0.85	677	C01	14488446	
	4	Bn-A01-p2688662	2.70E-05	7.74	27	A01	2194542	qA1-5(Wang et al.2015)^[^^11^^]^
	5	Bn-scaff_17423_1-p100318	0.000119	0.48	single marker	A09	NA^3^	
	6	Bn-A01-p15497190	4.41E-05	5	57	A01	12941895	qA1-5(Wang et al.2015)^[^^11^^]^
	7	Bn-Scaffold000178-p33587	1.95E-07	1.45	single marker	A09	NA^3^	
	8	Bn-scaff_15794_1-p347392	5.21E-41	25.67	775	C03	55942754	qC3-3(Wang et al.2015)^[^^11^^]^
	9	Bn-A04-p16528010	4.43E-05	0.47	288	A04	16689032	
	10	Bn-A04-p17358519	7.05E-07	2.44	single marker	C04	46402393	
	11	Bn-A06-p112339	4.94E-08	4.83	364	A06	NA^3^	
	12	Bn-Scaffold000217-p6025	8.40E-12	6.06	361	A05	22747105	
	13	Bn-scaff_16614_1-p373513	1.71E-11	0.59	single marker	NA^3^	NA^3^	
	14	Bn-A03-p2297079	7.65E-08	1.94	single marker	A03	1863826	
	15	Bn-A01-p28047872	1.18E-07	1.78	single marker	NA^3^	NA^3^	
	16	Bn-A08-p13239816	3.77E-08	21.16	510	A08	10991898	qA8-5(Wang et al.2015)^[^^11^^]^
	17	Bn-scaff_15699_1-p577914	4.02E-05	0.49	509	C08	16728901	
	18	Bn-A06-p23865356	2.19E-09	1.55	420	A06	22856806	
	19	Bn-A03-p8924852	1.16E-12	3.39	173	A03	8233061	
Glucosinolate	1	Bn-scaff_15747_1-p108596	2.11E-06	13	676	C01	14196095	TN-q.mcG-C1d(Feng et al.2012)^[^^9^^]^
	2	Bn-scaff_19168_1-p31612	4.39E-05	1.42	79	C01	36005339	
	3	Bn-A04-p12259499	1.21E-05	0.24	269	A04	13248240	TN-q.mcG-A4c(Feng et al.2012)^[^^9^^]^
	4	Bn-A04-p13930713	1.36E-07	1.16	single marker	A04	NA^3^	
	5	Bn-scaff_15918_1-p229987	3.20E-10	2.86	722	C02	42160463	TN-q.mcG-C2b(Feng et al.2012)^[^^9^^]^
	6	Bn-scaff_15794_1-p437864	1.45E-25	18.07	774	C03	55837809	TN-q.mcG-C3c(Feng et al.2012)^[^^9^^]^
	7	Bn-C14160250-p3687	2.05E-19	17.14	509	A08	NA^3^	
	8	Bn-A03-p7838070	2.94E-07	3.97	170	A03	7130138	TN-cqS-Aro-GST-A3a(Feng et al.2012)^[^^9^^]^
Total/average	151		1.94E-05	3.221133333				

^1^ More detailed information of each genetic bin is listed in S2 Table.

^2^ The physical position is presented by the start position of each SNP with unique position to the reference genome of *B*. *napus*, *Darmor-bzh 4*.*1*, and more information is also available in S2 Table.

^3^ Not available because of absent of alignment or multiple alignment positions.

The FDR (false-discovery rate) significance level is P<0.1for the detection of associated markers.

We used five-fold cross-validation to reliably estimate the potential of marker-assisted selection (MAS). The average accuracy of MAS ranged from 0.47 for protein content to 0.81 for erucic acid content (Table 3). These values were substantially lower compared to the non-cross-validated results (Table 2), underlining the need to validate findings of linkage mapping.

**Table 3:** Average prediction accuracy of four genomic selection methods and marker assisted selection (MAS) for six seed quality traits of the TN DH population.

Marker Type	Method	Oil content	Protein content	Erucic acid	Linolenic acid	Stearic acid	Gluco-sinolates	Average
13,678 SNPs	RR-BLUP	0.74	**0.65**	0.81	0.73	**0.78**	0.69	0.73
	BayesCπ	**0.75**	0.62	**0.89**	0.49	0.61	**0.79**	0.69
	EG-BLUP	0.72	0.62	0.79	**0.74**	0.75	0.67	0.72
	GBLUP	0.72	0.61	0.77	0.73	0.75	0.67	0.71
	MAS	0.52	0.47	0.81	0.51	0.74	0.64	0.62
1,527 representative SNPs*	RR-BLUP	**0.76**	**0.66**	0.83	0.75	**0.81**	0.72	0.76
	BayesCπ	**0.76**	0.64	**0.88**	0.45	0.61	**0.79**	0.69
	EG-BLUP	0.75	0.64	0.81	**0.76**	0.79	0.71	0.74
	GBLUP	**0.76**	0.64	0.82	0.75	0.8	0.72	0.75
	MAS	0.59	0.45	0.74	0.54	0.68	0.62	0.6
1,527 random SNPs*	RR-BLUP	0.72	0.63	0.81	0.72	0.77	0.69	0.72

*The 1,527 representative SNPs are specifically selected from the 1,527 individual genetic bins of the TN DH population, while the 1,527 random SNPs are randomly selected from the total 13,678 polymorphic SNPs of the TN DH population. Marker assisted selection (MAS) is based on markers significantly associated with the respective traits outlined in detail in the Material and Methods.

### Accuracies of genome-wide prediction in the TN DH population

We used four different models to investigate the efficiency of genome-wide prediction for the six seed quality traits. Genomic selection significantly showed higher prediction accuracies than MAS for all traits, with the most pronounced differences observed for linolenic acid, oil, and protein content (Table 3). The average prediction accuracy of RR-BLUP was the highest, while BayesCπ performed best for erucic acid and glucosinolate content. The most complex model comprising main and epistatic effects, EG-BLUP, performed best for linolenic acid content. In general, traits with high heritability could be predicted with higher accuracy compared to traits with low heritability.

As expected for a bi-parental mapping population, a large number of markers were in tight LD and could thus be grouped into genetic bins because of the absence of recombination events. We reduced the co-linearity among markers and removed redundant markers in full linkage disequilibrium, resulting in a subset of 1,527 SNP markers (S2 Table, S2 Fig). Prediction accuracy increased on average by 3% using the reduced 1,527 representative marker set compared to genomic selection based on all SNPs (Table 3).

### Effects of marker density, training population size, and number of environments on prediction accuracy

**Fig 3:**
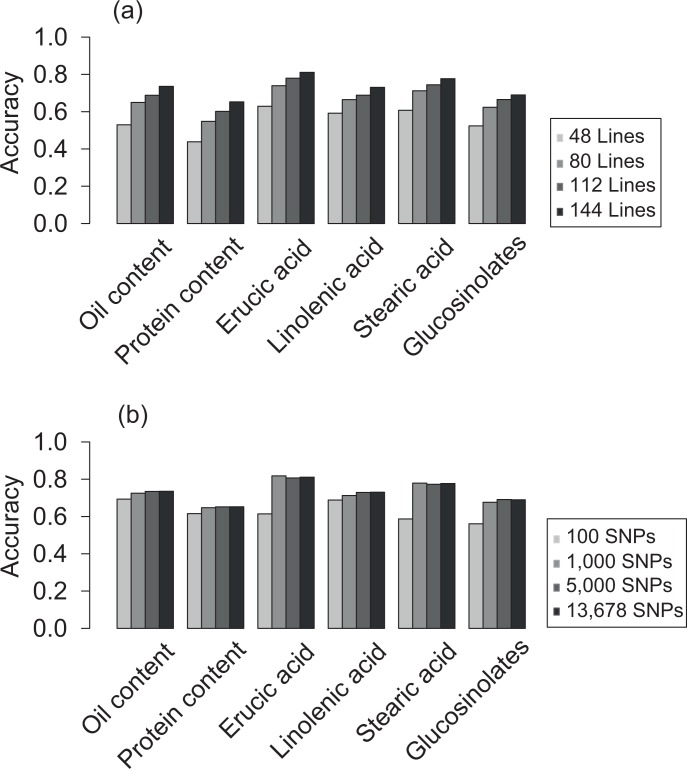
Average prediction accuracy of genomic selection applying RR-BLUP based on (a) varying training population sizes and (b) number of markers.

Genome-wide prediction based on RR-BLUP performed best on average and, in addition, was computationally efficient. Therefore, we conducted comprehensive analyses on the factors driving the accuracy in genome-wide prediction exclusively based on RR-BLUP. We varied the training population size and marker density and examined the accuracy of genome-wide predictions in our study. The accuracy remained in the range of 0.44 to 0.67 for all traits using only 48 lines as the training set (Fig 3). Interestingly, prediction accuracy reached a peak with 1,000 randomly selected markers and decreased only marginally for a subset of 100 markers. The prediction accuracy increased by ~4% for all six traits when using a representative set of markers compared to the 1,527 random evenly distributed markers (Table 2). Thus, our results indicated that to improve the accuracy of genome-wide prediction in a bi-parental population, the population size is more important than the density of markers.

**Fig 4:**
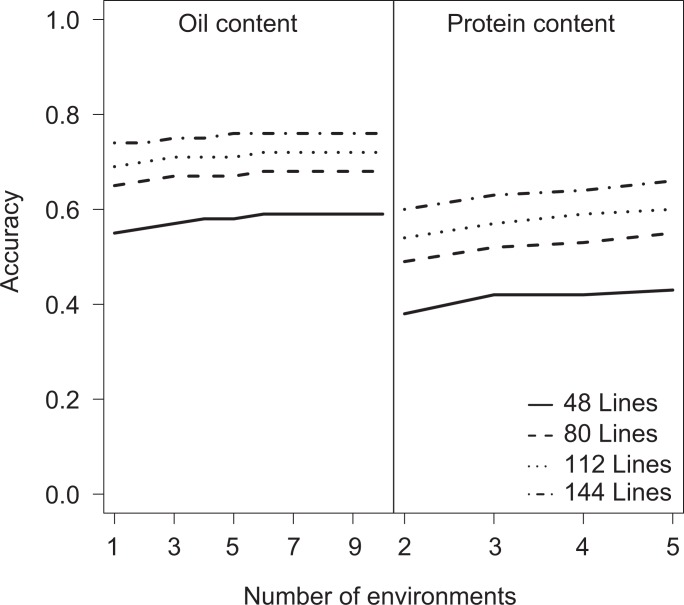
Prediction accuracy of oil content and protein content using marker data for 1,527 representative SNPs according to different numbers of environments and training set size.

We further studied the effects of the number of environments and training population size on the accuracy of genomic selection by focusing on oil and protein content. The traits were selected because oil content was evaluated in a large number of 11 environments and protein content exhibited a high heritability. We randomly selected training sets comprising n = 48, 80, 112, and 144 lines evaluated for oil content evaluated in subsets of environments (k = 2, 3,…, 11 for oil content; k = 2, 3, 4, 5 for protein content). The accuracy was estimated as the Pearson moment correlation coefficient between predicted genotypic values and the adjusted entry means of all remaining lines evaluated across all environments. This type of cross-validation allows for the study of the prediction accuracy assuming reduced phenotyping intensity. As the test set was not evaluated in any of the environments, their performance could not be estimated by phenotypic correlations between environments. The prediction accuracies based on phenotypic data from only two environments were 0.73 for oil content and 0.60 for protein content (Fig 4). Compared to the accuracy evaluated with the full dataset, the accuracy decreased only in the range of 3% to 6%. The accuracy remained at 0.55 for oil content when only 48 lines and 2 environments were used.

### Accuracies of genome-wide prediction for seed oil content and protein content validated in a diverse population of 117 *B*. *napus* lines

A panel of 117 diverse lines was genotyped and phenotyped in one environment in order to validate the prediction accuracies of seed oil content and protein content. A total of 1148 common genetic bin markers across the AC genome, were screened for the two populations. Since we observed the highest accuracies for RR-BLUP in the TN DH population, we also used this method for prediction. The prediction accuracy amounted to 0.14 for protein content and 0.17 for oil content based on the genetic bin markers.

## Discussion

### Erucic acid, stearic acid, and glucosinolate content are promising targets for marker-assisted selection

Understanding the genetic basis of seed oil yield and quality is important for efficient rapeseed breeding [10]. Previous studies revealed differences in the complexity of the genetic architecture of the six quality traits examined in our study [1, 3, 9–11, 17, 20, 22, 72], which were further substantiated using five-fold cross-validations (Table 2; S3 Table). Oil, protein, and linolenic content are characterized by the absence of a reliable large-effect QTL, while erucic acid, stearic acid, and glucosinolate content are to a large degree controlled by a few QTL exhibiting large effects. For instance, the major QTL located in A08 and C03 (Table 2) totally explained 76.66% of the phenotypic variance for erucic acid, which has been widely identified previously in TN DH population and other mapping populations of *B*. *napus* [21, 22, 24, 73]. The major QTL located on C03 and explaining 16.44% of the phenotypic variance for oil content was identified in both of TN DH population and KN DH population [74]. The QTL with large genetic effects for total seed glucosinolates located in A08, C01 and C03, were also identified previously in this and other mapping populations [9, 19, 75]. These QTLs are interesting targets for marker-assisted selection, which can be applied in rapeseed breeding in combination with the enrichment of target alleles for F_2_ populations prior to producing DH populations. Besides of the consistent identified QTL, we also detected several new QTLs accounting for these seed quality traits with minor effects in TN DH population compared to the previous QTL identification in this population [10, 11, 22], which possibly because of the improved detection power using the high density SNP markers compared to the previous QTL identification using the relatively low-density markers. For example, the QTL “Bn-Scaffold000217-p20168” in C05, “Bn-scaff_16130_1-p1013445” and “Bn-scaff_16130_1-p1039452” located in C07 was newly identified for seed oil content of this population compared to that detected in Jiang et al., (2014). It is important to note that due to the absence of a physical position of 2,828 SNPs without alignment to the reference genome, we could not compare those QTL without unique physical position with previous studies.

### Genetic architecture marginally impacts the choice of the genome-wide prediction model

Previous simulation studies revealed that equal shrinkage of marker effects as applied in RR-BLUP can be inappropriate for traits influenced by QTLs exhibiting large effects [13, 76]. In these cases, Bayesian models such as BayesB or BayesCπ, which allow specific shrinkage of every marker [77], are expected to outperform RR-BLUP. The superiority of BayesB over RR-BLUP has been reported for glucosinolate content in a previous genome-wide prediction study based on a diverse panel of 391 rapeseed lines derived from nine families [31]. Superiority of Bayes models versus RR-BLUP has also been observed for flowering time in the TN DH population [50]. In accordance with this observation, prediction accuracies for erucic acid and glucosinolate content were maximized when applying BayesCπ, with improvements of 8–10% compared to RR-BLUP (Table 2). In contrast, for stearic acid, RR-BLUP outperformed BayesCπ despite the presence of large-effect QTL. This is most likely due to two reasons. First, the ratio between the phenotypic variance explained by the two large-effect QTL versus that explained by the remaining small-effect QTL is approximately 1 to 1 for stearic acid content, while this ratio is 5 to 1 for erucic acid and glucosinolate content. Second, one large-effect QTL controlling stearic acid is reflected in several marker-trait associations with SNPs being in tight linkage disequilibrium (*r*^2^ >0.8), while the QTLs are reflected by only a limited number of SNPs for erucic acid and glucosinolate content.

Epistasis, the interaction between genes [78], is an additional potential force influencing the choice of the biometrical model for genome-wide prediction [65]. Previous linkage and linkage disequilibrium mapping studies in rapeseed indicated that epistatic effects are involved in fatty acid metabolism [11, 47]. Consequently, we implemented EG-BLUP for genome-wide prediction, which explicitly considers digenic additive by additive epistatic effects [65]. We observed, however, higher prediction accuracies of EG-BLUP compared to the other genome-wide prediction models only for linolenic acid content (Table 2). Moreover, the gains in prediction accuracy were only marginal. These negligible benefits are in contrast to the non-cross-validated results of previous linkage and linkage disequilibrium studies [11, 47] and point to the strong need to validate the role of epistatic effects. In summary, the accuracy of genomic selection does not crucially depend on the choice of a suitable genome-wide prediction model and is an attractive alternative to marker-assisted selection.

### Implementation of genome-wide prediction in rapeseed breeding

The successful implementation of genome-wide prediction in rapeseed breeding requires that a certain threshold of prediction accuracy is realized [40, 79]. Previous model studies in wheat and maize suggested a threshold for the prediction accuracy of 0.5 [80, 81]. We chose two important traits, oil content and protein content, to illustrate the size of the training population, the number of environments, and the marker density required to reach a prediction accuracy of 0.5 for the bi-parental population.

In accordance with previous studies based on bi-parental populations [82–85], approximately one thousand markers were required before the prediction accuracy plateaued (Fig 3). Increasing the number of markers introduced problems due to collinearities. Prediction accuracies were higher for a reduced a set of 1,527 SNPs, which represented recombination loci in the population, in contrast to the full 13,678-marker set (Table 3). Thus, to improve the accuracy of genome-wide prediction in a bi-parental population, the population size indicating recombination events obtained is more important than the density of markers.

The number of lines has a greater impact on the prediction accuracy than the number of environments (Figs 3 and 4). The prediction accuracy is already stagnating at three environments, and thus it is more efficient to invest in training population size. For protein content, approximately 144 lines evaluated in two environments were needed to reach an accuracy of 0.6. For oil content, prediction accuracy amounted to 0.6 when the training population was decreased to 80 lines and the number of environments reduced to two. These results suggest that genome-wide prediction can be successfully implemented in bi-parental populations even with small training population sizes and is an attractive complement to phenotypic selection to improve seed quality traits.

The prediction accuracy within bi-parental populations is of central importance examining the potential to implement genome-wide prediction in breeding programs exploiting the double-haploid technology. Moreover, it is of interest to study the potential to use the prediction model also in unrelated populations. We examined an extreme validation scenario for the prediction of seed oil and protein content using a genetically diverse sample of 117 lines which were based on crosses between *B*. *rapa* and *B*. *carinata* accessions [55, 56]. The prediction accuracy in this independent and genetically very distinct validation population still amounted to 0.14 for protein content and 0.17 for oil content. While interpreting the prediction accuracies it has to be considered that the validation population exhibits genome segments from *B*. *rapa*/*B*. *carinata*. However, the used *Brassica* 60K-SNP array was developed based on the AC genome sequence of *B*. *rapa*, *B*. *olearaca* and *B*. *napus*. Thus, the lack of unique polymorphisms of *B*. *carinata* is expected to impair the prediction accuracies. Taking this into consideration, our independent validation reflects the high quality of the developed calibration models even in very diverse backgrounds highlighting the prospects of genome-wide prediction for routine rapeseed breeding programs.

## Supporting Information

S1 Fig Quantile-quantile plots of association mapping for six traits using different methods. The green lines are the -log_10_ P-values of the linear regression method. The red lines are the -log_10_ P-values of the stepwise multiple linear regression method. The expected uniform distribution of negative -log_10_ P-values is indicated by the diagonal line in blue.
(PDF)

S2 Fig Decay of linkage disequilibrium with physical distance. Within each physical distance class, marker pairs are clustered into five groups with varying r^2^ values.
(JPEG)

S1 Table Locations, years and environments for the field experiment. (DOCX)

S2 Table The physical alignment information of the SNPs of the TN DH population to the reference "Darmor-bzh" genome of *B*. *napus*. (XLSX)

S3 Table Summary of the phenotypic data of six quality traits assessed in the TN DH population across environments. (XLSX)

## Abbreviations

QTL: quantitative trait loci
DH: diploid haploid
BLUEs: best linear unbiased estimates
LD: linkage disequilibrium
FDR: false-discovery rate
PVE: phenotypic variance explained by all QTL
GBLUP: genomic best linear unbiased prediction
RR-BLUP: ridge regression best linear unbiased prediction
EG-BLUP: extended GBLUP model
