# Supplementary figures and images for: Seed Quality Traits Can Be Predicted with High Accuracy in Brassica napus Using Genomic Data
Source: PLoS One. 2016 Nov 23;11(11):e0166624. doi: 10.1371/journal.pone.0166624 (PMC5120799; doi:10.1371/journal.pone.0166624)

**Oil content**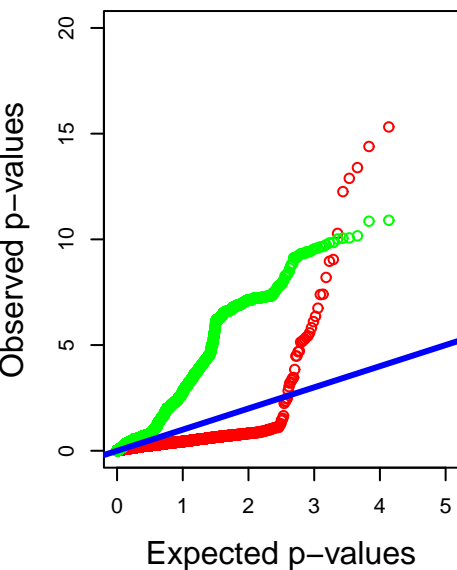**Protein content**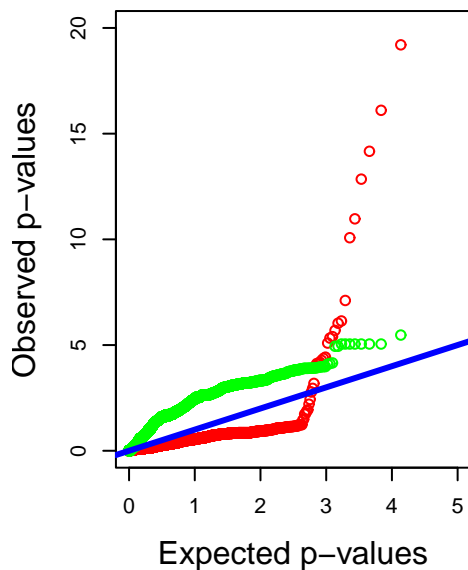**Erucic acid**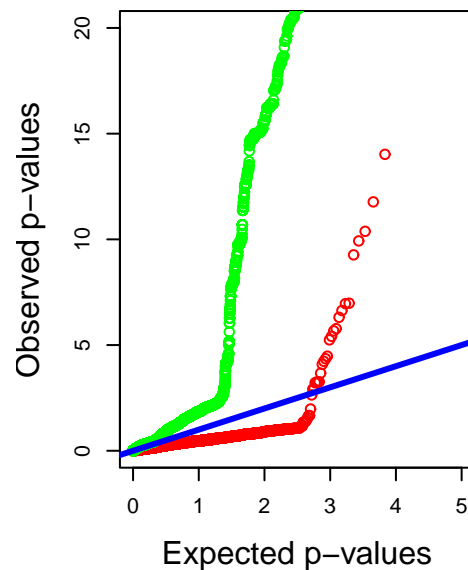**Linolenic acid**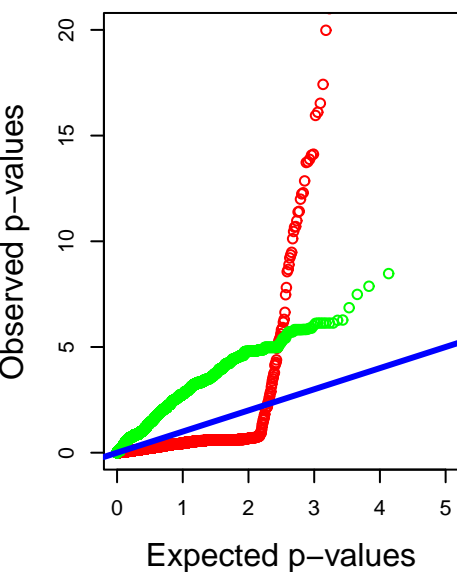**Stearic acid**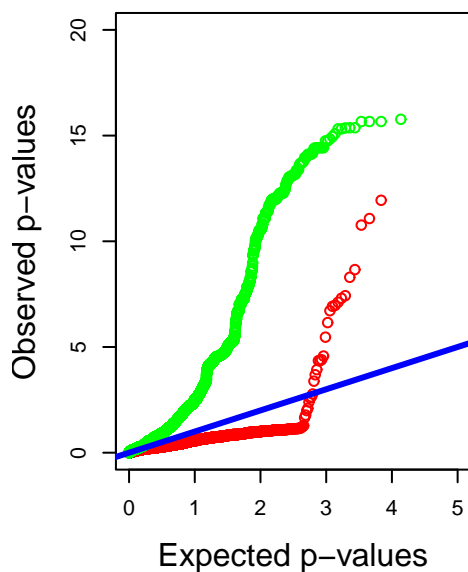**Glucosinolates**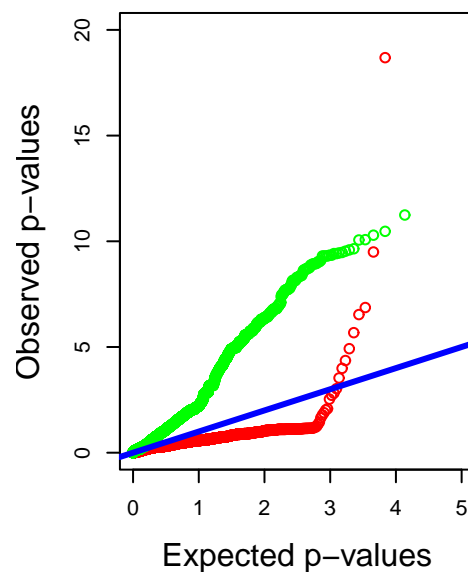

Supplement: S1 Fig — The green lines are the -log10 P-values of the linear regression method. The red lines are the -log10 P-values of the stepwise multiple linear regression method. The expected uniform distribution of negative -log10 P-values is indicated by the diagonal line in blue. (PDF) [file pone.0166624.s001.pdf]

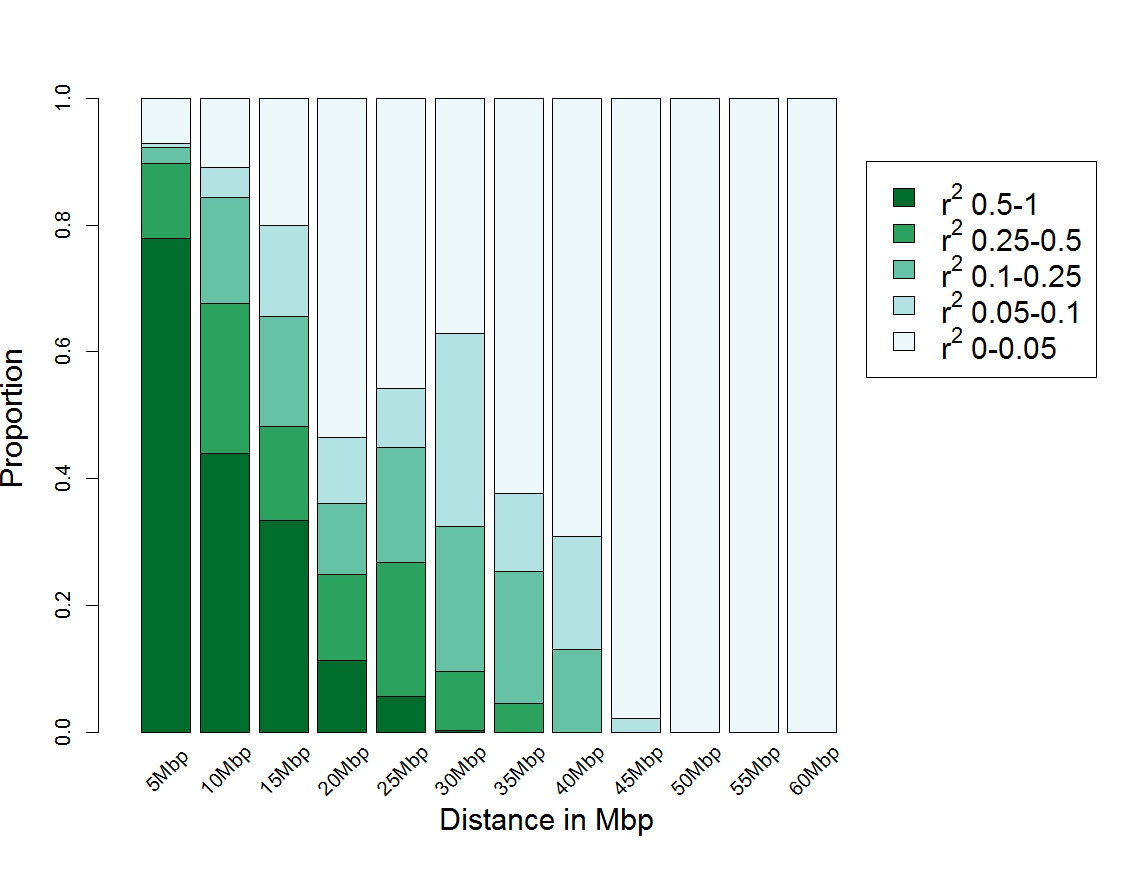

Supplement: S2 Fig — Within each physical distance class, marker pairs are clustered into five groups with varying r2 values. (JPEG) [file pone.0166624.s002.jpeg]
