# Supplementary material for: Seed Quality Traits Can Be Predicted with High Accuracy in Brassica napus Using Genomic Data
Source: PLoS One. 2016 Nov 23;11(11):e0166624. doi: 10.1371/journal.pone.0166624 (PMC5120799; doi:10.1371/journal.pone.0166624)
Supplement: S1 Table — (DOCX) [file pone.0166624.s003.docx]

**S1 Table.** **Locations, years and environments for field experiment.**

| Location | Year | Environment |
| --- | --- | --- |
| Hangzhou, Zhejiang, E120°12′/ N30°16′/ 40m | Oct,2006 - May,2007 | E7 |
| Dali, Shaanxi, E109°56′/ N34°52′/ 800m | Sep,2002 - Jun,2003 | N3 |
| Dali, Shaanxi, E109°56′/ N34°52′/ 800m | Sep,2003 - Jun,2004 | N4 |
| Dali, Shaanxi, E109°56′/ N34°52′/ 800m | Sep,2005 - Jun,2006 | N6 |
| Dali, Shaanxi, E109°56′/ N34°52′/ 800m | Sep,2006 - Jun,2007 | N7 |
| Wuhan, Hubei, E114°21′/ N30°37′/ 40m | Oct,2002 - May,2003 | S3 |
| Wuhan, Hubei, E114°21′/ N30°37′/ 40m | Oct,2003 - May,2004 | S4 |
| Jiangling, Hubei, E113°25′/N30°30′/ 40m | Oct,2004 - May,2005 | S5 |
| Daye, Hubei, E114°48′/ N30°06′/ 100m | Oct,2005 - May,2006 | S6 |
| Wuhan, Hubei, E114°21′/ N30°37′/ 40m | Oct,2005 - May,2006 | S6A |
| Wuhan, Hubei, E114°21′/ N30°37′/ 40m | Oct,2006 - May,2007 | S7 |
